# Abnormal synergies and associated reactions post-hemiparetic stroke reflect muscle activation patterns of brainstem motor pathways

**DOI:** 10.3389/fneur.2022.934670

**Published:** 2022-10-10

**Authors:** Laura M. McPherson, Julius P. A. Dewald

**Affiliations:** ^1^Program in Physical Therapy, Washington University School of Medicine, St. Louis, MO, United States; ^2^Department of Neurology, Washington University School of Medicine, St. Louis, MO, United States; ^3^Program in Neurosciences, Division of Biology and Biomedical Sciences, Washington University School of Medicine, St. Louis, MO, United States; ^4^Department of Biomedical Engineering, Northwestern University, Chicago, IL, United States; ^5^Department of Physical Therapy and Human Movement Sciences, The Feinberg School of Medicine, Northwestern University, Chicago, IL, United States; ^6^Department of Physical Medicine and Rehabilitation, The Feinberg School of Medicine, Northwestern University, Chicago, IL, United States

**Keywords:** stroke, upper limb, rehabilitation, flexion synergy, extension synergy, associated reaction, brainstem motor pathways

## Abstract

Individuals with moderate-to-severe post-stroke hemiparesis cannot control proximal and distal joints of the arm independently because they are constrained to stereotypical movement patterns called flexion and extension synergies. Accumulating evidence indicates that these synergies emerge because of upregulation of diffusely projecting brainstem motor pathways following stroke-induced damage to corticofugal pathways. During our recent work on differences in synergy expression among proximal and distal joints, we serendipitously observed some notable characteristics of synergy-driven muscle activation. It seemed that: paretic wrist/finger muscles were activated maximally during contractions of muscles at a different joint; differences in the magnitude of synergy expression occurred when elicited via contraction of proximal vs. distal muscles; and associated reactions in the paretic limb occurred during maximal efforts with the non-paretic limb, the strength of which seemed to vary depending on which muscles in the non-paretic limb were contracting. Here we formally investigated these observations and interpreted them within the context of the neural mechanisms thought to underlie stereotypical movement patterns. If upregulation of brainstem motor pathways occurs following stroke-induced corticofugal tract damage, then we would expect a pattern of muscle dependency in the observed behaviors consistent with such neural reorganization. Twelve participants with moderate-to-severe hemiparetic stroke and six without stroke performed maximal isometric torque generation in eight directions: shoulder abduction/adduction and elbow, wrist, and finger flexion/extension. Isometric joint torques and surface EMG were recorded from shoulder, elbow, wrist, and finger joints and muscles. For some participants, joint torque and muscle activation generated during maximal voluntary contractions were lower than during maximal synergy-induced contractions (i.e., contractions about a different joint), particularly for wrist and fingers. Synergy-driven contractions were strongest when elicited via proximal joints and weakest when elicited via distal joints. Associated reactions in the wrist/finger flexors were stronger than those of other paretic muscles and were the only ones whose response depended on whether the non-paretic contraction was at a proximal or distal joint. Results provide indirect evidence linking the influence of brainstem motor pathways to abnormal motor behaviors post-stroke, and they demonstrate the need to examine whole-limb behavior when studying or seeking to rehabilitate the paretic upper limb.

## Introduction

Stereotypical movement patterns that emerge in the upper limb of individuals with moderate-to-severe post-stroke hemiplegia present a substantial barrier to completing functional tasks because they interfere with the ability to control proximal and distal joints independently. These obligatory movement patterns are described clinically as the flexion synergy (shoulder abduction coupled with elbow, wrist, and finger flexion) and the extension synergy (shoulder adduction coupled with elbow extension, wrist flexion or extension, and finger flexion) (1–5). They emerge as a result of an increased influence of diffusely projecting brainstem motor pathways following stroke-induced damage to the corticospinal pathway (6–10).

Over the last decade, we have extensively characterized the flexion and extension synergies at the shoulder, elbow, wrist, and fingers (1, 4, 5), extending the laboratory's previous work that focused on the proximal manifestation of the synergies at the shoulder and elbow joints (3, 11–15). Recently, we found that some characteristics of flexion and extension synergy expression differ among shoulder, elbow, wrist, and finger muscles (1), expanding our growing body of knowledge that provides the foundation for the development of targeted rehabilitation strategies. During the analysis of data from that study (1), we serendipitously observed some additional characteristics of synergy-driven muscle activation that occurred frequently enough to warrant further exploration.

First, we noticed that paretic wrist and finger muscles seemed to be activated maximally while individuals contracted muscles at a different joint, not during a maximal voluntary contraction of the wrist and finger muscles themselves, as is typical. Second, there seemed to be differences in the magnitude of synergy expression when it was elicited via contraction of proximal muscles vs. distal muscles. Third, there seemed to be consistent movement resembling flexion or extension synergy patterns in the paretic limb during maximal efforts with the non-paretic limb (a phenomenon described clinically as an associated reaction). The strength of these associated reactions appeared to differ based on which muscles in the non-paretic limb were being activated. We did not formally investigate these observations for inclusion in that study.

Thus, the goals of this article were to formally investigate the observations, specifically focusing on differences between proximal vs. distal joints and flexor vs. extensor muscles, and to interpret them within the context of the neural mechanisms thought to underlie stereotypical movement patterns. If upregulation of brainstem motor pathways occurs following stroke-induced corticospinal and corticobulbar tract damage, then we would expect a pattern of muscle dependency in the observed behaviors consistent with the ways in which the muscles are impacted by such neural reorganization. Specifically, while all upper limb muscles are controlled by both the precise, sophisticated lateral corticospinal system and the diffusely projecting, comparatively more crude brainstem motor system, the two motor systems have different contributions to neural control of proximal vs. distal muscles. Brainstem motor pathways have the strongest projections to proximal muscles (16–18), which is in line with their role in postural stability. Conversely, the corticospinal pathway has the strongest and most frequent projections to distal muscles (19), which is in line with their role as the predominant muscles for fine motor control. In addition, the reticulospinal pathway, which is the brainstem pathway thought to underlie the flexion synergy (6–10, 14), has bilateral effects in the upper limbs and favors the facilitation of flexor muscles on the ipsilateral side (16, 20–23). Following corticospinal and corticobulbar damage, activity of brainstem pathways may be inadequately balanced due to the reduced activity of the corticospinal tract and/or a loss of oligosynaptic inhibitory cortico-reticular connections (24). As a result, the way that muscles are activated may reflect characteristics of brainstem pathways.

Based on this framework, our study had the following aims and specific predictions. The first aim of the study was to determine which paretic upper limb muscles are activated maximally during contractions of muscles at other joints (i.e., during elicitation of the flexion and extension synergies) rather than during voluntary contractions of the muscles themselves. We predicted that maximal activation of proximal muscles (i.e., those of the shoulder and elbow) would be achieved through voluntary contractions but that maximal activation for the most distal muscles (i.e., those of the wrist and fingers) would occur during synergy-driven contractions.

The second aim of the study was to determine whether the magnitude of flexion and extension synergy expression differs when elicited via maximal contractions of proximal vs. distal muscles in the paretic arm. Because proximal muscles are more heavily innervated by brainstem pathways than distal muscles, we predicted that activation of proximal muscles would result in stronger synergy expression compared with activation of distal muscles.

The third aim of the study was to determine whether the magnitude of associated reactions differs when elicited via maximal contractions of proximal vs. distal muscles of the *contralateral* (non-paretic) arm. We predicted that activation of proximal muscles, compared with activation of distal muscles, would result in stronger associated reactions, and that the associated reactions would be stronger in flexor compared with extensor muscles. This is because reticulospinal pathways have stronger bilateral projections to proximal compared to distal muscles and flexor compared to extensor muscles.

Our findings were consistent with our predictions. For some participants, joint torque and muscle activation generated during maximal voluntary contractions were lower than during maximal synergy-induced contractions. This was more prevalent and more severe in magnitude at the wrist and fingers than at the shoulder and elbow. Synergy-driven contractions were strongest when elicited via proximal joints and weakest when elicited via distal joints. Associated reactions in the paretic wrist/finger flexors were stronger than those of other paretic muscles and were the only ones whose response depended on whether the contralateral contraction was at a proximal or distal joint.

All data utilized in the analyses presented here were collected as part of the same experiment protocol, which included both ipsilateral and contralateral contractions (see Methods). Some analyses of data from the ipsilateral contractions were reported in McPherson and Dewald (1). Portions of our findings have been reported in the abstract (25) and dissertation (26) forms.

## Methods

### Participants

Individuals with chronic hemiparetic stroke (>1 year prior) were recruited through a departmental research database. Participation required enough passive range of motion at the shoulder, elbow, wrist, and fingers to be placed comfortably in the isometric testing setup (described in the following subsection). A physical therapist performed a clinical exam on potential participants. Clinical motor deficits that were accepted for inclusion in the study were those consistent with cortical or sub-cortical lesions (e.g., unilateral hemiparesis with non-cerebellar, non-brainstem clinical signs). We could obtain specific lesion locations for nine of the 12 participants from either their medical records, or when available, a computed tomography scan and/or a T1-weighted magnetic resonance imaging scan. For scans that had not been interpreted by a radiologist, research personnel with training in neuroanatomy identified lesion locations. Exclusion could result from any one of the following four conditions: first, an upper extremity Fugl-Meyer Motor Assessment (FMA) (27) score outside of the 10–44 range (0–9 indicating near paralysis and 45–66 indicating mild impairment); second, a Chedoke-McMaster Stroke Assessment hand portion (CMSAh) (28) score >5 (indicating mild impairment); third, significant impairment of vision or upper extremity tactile somatosensation; or fourth, the use of botulinum toxin in the paretic upper limb within 3 months.

Twelve individuals with chronic post-stroke hemiparesis met all inclusion criteria and completed the study (three females, nine males; mean age ± SD: 59.0 ± 6.2 years, range 47–70; mean ± time post-stroke 10.3 ± 5.9 years; range: 3.5–26.6; Table 1). Participants exhibited severe-to-moderate upper limb motor impairment according to the FMA with scores ranging from 13 to 31 of 66 possible (mean: 23.0). They also exhibited severe-to-moderate hand motor impairment according to the CMSAh with a score ranging from 2 to 4 of a possible 7 (mean: 3.0). Seven participants had right-sided hemiparesis and five had left-sided hemiparesis.

**Table 1:** Demographics of the participants with chronic hemiparetic stroke.

**Participant**	**Sex**	**Affected/dominant**	**Lesion location**	**Yrs post-stroke**	**FMA**	**CMSAh**
1	F	R/R	BG, TH, IC	26.6	20	3
2	M	L/L	N/A	11.4	24	2
3	M	R/R	BG, IC, CFL, SFL, IN	4.5	13	3
4	F	R/R	BG, IC, TH, HC	5.3	30	3
5	M	L/L	IC	3.8	25	4
6	M	L/R	BG, IC	6.5	24	3
7	F	L/R	N/A	9.0	15	3
8	M	R/L	BG, TH, IC, CTL	17.0	19	2
9	M	R/L	N/A	25.5	26	3
10	M	L/L	CPL, CFL, CTL	5.6	31	3
11	M	R/L	IC	5.1	20	4
12	M	R/R	IC	3.5	29	3
Mean (SD)				**10.3 (8.3)**	**23.0 (5.7)**	**3.0 (0.6)**

FMA, Upper extremity score of the Fugl-Meyer Motor Assessment (max = 66); CMSAh, Hand portion of the Chedoke-McMaster Stroke Assessment (max = 7); BG, basal ganglia; TH, thalamus; IC, internal capsule; CFL, cortical frontal lobe; SFL, sub-cortical frontal lobe; CPL, cortical parietal lobe; HC, hippocampus; IN, insula; N/A, not available.

Six control participants without known neurological injury (four males, two females; mean age 60.6 years) were included for comparison with the non-paretic limb of participants with stroke. All participants gave informed consent for participation in the study, which was approved by the Institutional Review Board of Northwestern University.

### Experimental setup and data collection

**Figure 1:**
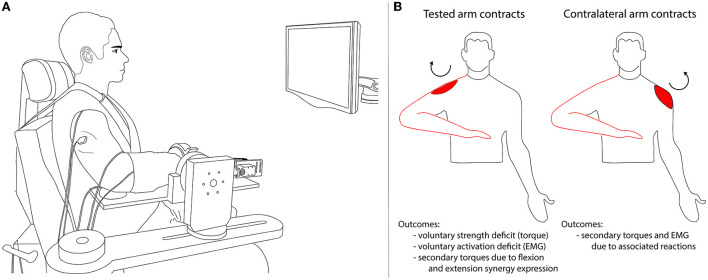
Experimental setup **(A)** and overview of the experimental protocol and outcome variables **(B)**. In **(A)** the participant performed isometric torque generation in one direction per trial (shoulder abduction or adduction, elbow flexion or extension, wrist flexion or extension, or finger flexion or extension). Secondary torques generated at joints other than the intended (primary) torque direction were recorded along with surface EMG of shoulder, elbow, wrist, and finger muscles. In **(B)** the tested arm that was secured to the isometric testing setup is outlined in red. The ipsilateral contraction conditions are illustrated on the left: the experimental task was completed by the tested arm. The contralateral contraction conditions are illustrated on the right: the experimental task was completed by the arm contralateral to the tested arm. The circular arrow and red shape indicate the primary torque direction and associated agonist muscle (in this example, shoulder abduction and deltoid).

The experimental protocol was conducted in a testing device capable of measuring isometric shoulder, elbow, wrist, and finger (i.e., metacarpophalangeal) joint torques simultaneously (1, 29) (Figure 1A). Participants were seated in an experimental chair (Biodex, Inc.) with shoulder/waist restraints to prevent shoulder girdle and trunk motion. The tested forearm was placed in a fiberglass cast to interface the arm rigidly with a six degree of freedom load cell (JR3, model 45E15A) through a Delrin ring. The wrist and fingers were placed in a custom Wrist and Finger Torque Sensor (WFTS) (30). The arm was positioned in 75° shoulder abduction, 40° shoulder flexion, 90° elbow flexion, 15° pronation, and 0° wrist flexion/extension. Paretic finger joints (i.e., metacarpophalangeal) were positioned at 15° finger flexion to accommodate a range of motion restrictions. The non-paretic and control finger joints had to be positioned at 0° finger flexion/extension because the increased strength of these groups slightly deformed the WFTS attachment bracket such that it would interfere with the isometric device if positioned at 15° of finger flexion. The contralateral (non-tested) arm rested comfortably at each participant's side. A computer monitor displayed real-time visual feedback of joint torque data.

Electromyographic (EMG) activity was recorded using active differential surface electrodes with a 1-cm interelectrode distance (16-channel Bagnoli EMG System; Delsys, Inc.; 1,000x gain, 20–450 Hz bandpass). On the tested arm, electrodes were placed over the following muscles according to the landmarks described by Perotto and Delagi (31): anterior deltoid (DELT), sternocostal head of the pectoralis major (PEC), biceps brachii (BIC), lateral head of the triceps brachii (TRI), extrinsic wrist and finger flexors (flexor carpi radialis [FCR], flexor digitorum profundus [FDP]), an intrinsic finger flexor (first dorsal interosseous [FDI]), extrinsic wrist and finger extensors (extensor carpi radialis [ECR], extensor digitorum communis [EDC]), and two thumb muscles (flexor pollicis brevis [FPB] and extensor pollicis longus [EPL]). The ground electrode was placed on the acromion of the scapula. In addition, electrodes were placed on the DELT, BIC, TRI, FCR, and ECR muscles of the contralateral arm. A signal conditioner (Frequency Devices, Model 9064) filtered (eighth order Butterworth low-pass filter, 500 Hz) and amplified EMG and wrist and finger torque data before digitization at a sampling frequency of 1 kHz. A handgrip dynamometer collected maximum grip forces used for descriptive purposes.

### Experimental protocol

A schematic of the experimental protocol is shown in Figure 1B.

#### Ipsilateral contractions

Each participant's maximum voluntary torques (MVTs) and corresponding maximum voluntary muscle contractions (MVCs) were measured in the tested arm during isometric torque generation in the following directions: shoulder abduction (SABD), shoulder adduction (SADD), elbow flexion (EF), elbow extension (EE), wrist flexion (WF), wrist extension (WE), finger flexion (FF), finger extension (FE), combined wrist and finger flexion, combined wrist and finger extension, thumb flexion (for FPB MVC only), and thumb extension (for EPL MVC only). Each maximal contraction lasted ~5 s. MVT directions were randomized, and trials within a direction were repeated until three trials with peak torque within 85% of the maximum torque value were obtained. If the last trial produced the largest peak torque, additional trials were collected. Participants were given vigorous verbal encouragement throughout MVT trials.

Visual feedback of torque in the target direction was shown except for during the WE and FE tasks while testing the paretic limb. Because most participants with stroke had little-to-no voluntary WE or FE on the paretic side, efforts to produce these movements often resulted in flexion [a phenomenon described previously (1, 5, 32)]. Therefore, no visual feedback was given for these directions to ensure participants' maximal effort.

#### Contralateral contractions

Maximum voluntary efforts in SABD, EF, EE, WF, and WE directions were performed for the arm contralateral to the tested arm. A physical therapist stabilized the contralateral arm at the participant's side, in a position of approximately 20° SABD, 90° EF, and 0° of wrist flexion/extension. The physical therapist manually resisted the contralateral arm during the maximal efforts, and the response of the tested arm was measured in the isometric testing device. For WF and WE directions, the forearm was fully pronated, and for the remaining directions, the forearm was in 0° pronation/supination.

### Data analysis

All data analyses were performed using custom MATLAB software. We converted the forces and moments collected from the six degree of freedom load cell attached at the forearm into shoulder and elbow joint torques using a Jacobian-based transformation matrix based on the geometry of the upper limb (i.e., limb segment lengths and joint angles) (3). See Sánchez et al. (33) and Goyal et al. (34) for detailed descriptions of a similar transformation matrix used for the lower extremity.

Torque and full-wave rectified EMG data were smoothed using an acausal one-sided moving average filter of window length 250 ms, baseline corrected so that any muscle tone at rest would not factor into subsequent analyses, and normalized to the largest value obtained over all of the ipsilateral contractions. We chose this normalization value instead of the maximum value during a voluntary contraction because paretic limb voluntary wrist and finger torque and EMG values were often very small in comparison to values generated during other MVT directions, resulting in inflated values when normalized.

For each MVT trial in the tested arm, maximal torque in the primary (intended) direction was determined. Secondary torques (i.e., those in degrees of freedom other than the primary direction) at the time of maximal primary torque were collected, as were EMG values at 50 ms preceding the maximal value, to account for an estimate of the electromechanical delay inherent to skeletal muscle (35).

To compare muscle activation during voluntary vs. synergy-driven contractions, we calculated a “voluntary activation deficit” as follows. We divided the maximal EMG value obtained during maximal voluntary contractions (i.e., when the muscle performed as an agonist, e.g., during finger flexion for FCR and FDP) by the maximal EMG value obtained during maximal contractions in all torque directions. We then subtracted this ratio from a value of 1 so that low values would indicate a small deficit in activation during voluntary contractions and high values would indicate a large deficit in activation during voluntary contractions, and it was multiplied by 100 to achieve a percentage. A value of 0% indicates a muscle's largest EMG value occurred during a voluntary contraction, and a value of 90% indicates a muscle's EMG value during voluntary contraction is 10% of the EMG value obtained during the largest synergy-driven contraction. We made the same calculation with torque data to compute the “voluntary strength deficit.”

To calculate the magnitude of synergy expression that resulted from each primary torque direction, mean synergy-driven torque was calculated by averaging the magnitude of all secondary torques.

For each MVT trial performed in the contralateral arm, maximal EMG of the agonist muscle was determined and EMG from the tested arm at that time point was collected.

### Statistical analysis

For statistical analyses, *p* ≤ 0.05 was used to determine significance. Values in the text are presented as mean ± SEM unless otherwise specified; values in Table 2 are presented as mean ± SD.

**Table 2:** Maximum voluntary strength measurements.

	**Maximal voluntary strength in primary direction**			**Participants in paretic group with synergy-driven strength greater than voluntary strength**		
	**Control**	**Non-Paretic**	**Paretic**	**Number**	**Mean (range) percent increase from voluntary to synergy-driven**	**Direction eliciting maximum torque**
SABD	50.1 ± 21.1	37.3 ± 17.5	*24.1 ± 13.2	0	—	—
SADD	67.8 ± 21.8	49.0 ± 23.2	*24.9 ± 11.6	2	18.6 (4.0–33.3)	EE x 2
EF	58.8 ± 18.6	51.3 ± 21.4	*24.0 ± 11.8	3	7.1 (1.0–16.5)	SABD x 3
EE	47.5 ± 16.0	38.1 ± 15.3	*18.5 ± 8.4	1	11.7	SADD
WF	18.5 ± 4.5	15.1 ± 5.6	*3.9 ± 1.8	5	48.5 (9.2–102.1)	SABD x 3, EF x 2
WE	8.6 ± 2.1	6.8 ± 2.2	—	—	—	—
FF	11.3 ± 2.7	7.7 ± 3.3	*3.0 ± 1.5	5	37.6 (9.0–103)	SABD x 1, SADD x 1, EF x 3
FE	2.1 ± 0.5	2.1 ± 0.7	—	—	—	—
Grip	417.3 ± 108.9	376.8 ± 115.7	*62.0 ± 28.0	—	—	—

Values are group mean ± standard deviation. Strength data are displayed in Newton-meters, except for grip strength which is displayed in Newton. An asterisk indicates a significant difference between paretic and non-paretic groups, and p-values are reported in the text.

To compare maximal voluntary strength between paretic and non-paretic limbs, we used a 2 × 7 linear mixed effects model to test the effects of the fixed, repeated factors of limb (paretic, non-paretic) and primary torque direction (SABD, SADD, EF, EE, WF, FF, Grip) as well as the limb-by-primary torque direction interaction on maximum voluntary torque (or force, in the case of grip strength) (GraphPad Prism, v8). Participant was included as a random factor, and the Greenhouse–Geisser correction was applied. The WE and FE directions were not included in this model because none of the paretic limbs could generate voluntary torque in these directions. To compare maximal voluntary strength between non-paretic and control limbs, we used a 2 × 9 linear mixed effects model to test the effects of the fixed factors of limb (non-paretic, control) and primary torque direction (repeated; SABD, SADD, EF, EE, WF, FF, WE, FE, Grip) as well as the limb x primary torque direction interaction on maximum voluntary torque/force (GraphPad Prism, v8). Participant was included as a random factor, and the Greenhouse–Geisser correction was applied. The effects of interest for both of the above models were the main effect of limb and the limb-by-direction interaction. Planned comparisons on the interactions using Fisher's least square difference tests determined differences between limbs for each primary torque direction.

To evaluate whether the voluntary strength deficit in torque differed among primary torque directions (i.e., SABD, SADD, EF, EE, WF, FF), we used a one-way repeated measures Friedman test (due to non-normally distributed data per the Shapiro–Wilk test).

To evaluate whether the voluntary activation deficit in EMG differed among muscles, we used a one-way repeated measures linear mixed effects model with a random factor of participant (GraphPad Prism, v8). Then, we conducted planned comparisons using an uncorrected Dunn's test to determine whether voluntary activation deficit values for each muscle differed from those for the deltoid. Out of 156 data points (12 participants × 13 muscles), there were five instances of missing data due to poor signal quality: one from the ECR, two from the FPB, and two from the EPL.

To determine differences in secondary torque generation between paretic and non-paretic limbs for each primary torque direction, we used a 2 × 8 repeated measures ANOVA (GraphPad Prism, v8) to test the main effect of limb and the limb-by primary torque direction interaction on joint torque. We used planned comparisons on the interaction using Fisher's least square difference tests to determine differences between limbs for each torque direction.

To determine whether there are differences in the strength of synergy expression for proximal-to-distal and distal-to-proximal joint combinations, we used a one-way ANOVA to test for differences among joint combinations for joints that elicited the flexion synergy. Then, we conducted planned comparisons using Fisher's least square difference tests among the salient joint combinations (see Results). We repeated these tests with data from joints that elicited the extension synergy. We also used a one-way ANOVA to test for differences in the strength of overall synergy expression for each primary torque direction, followed by planned comparisons that compared data from each primary torque direction with that of the SABD direction.

To examine the effect of contralateral muscle contractions on the tested arm, we averaged EMG data from the three wrist and finger flexors (FCR, FDP, FDI) to establish an EMG value for the wrist/finger flexor muscle group as a whole for brevity. In the same way, we established EMG values for the wrist/finger extensor muscle group by averaging EMG values from the ECR and EDC. To test differences between paretic and non-paretic limbs for each muscle group (wrist/finger flexors, wrist/finger extensors, biceps, triceps), our model included fixed factors of limb, contraction direction, and the limb-by-contraction direction interaction [R, version 3.6.3 with lme4 package (36)]. A random intercept was included, clustered by participants. To test differences in flexor and extensor muscle groups within the paretic limb, our model included fixed factors of the muscle group, contraction direction, and the muscle group-by-contraction direction interaction. A random intercept and random slopes of muscle group and torque direction were included (clustered by participants). Estimation of fixed and random effects for all models using restricted maximum likelihood estimation.

## Results

Maximum voluntary strength measurements from the paretic, non-paretic, and control limbs are shown in Table 2. The paretic limb was significantly weaker than the non-paretic limb in all directions (main effect of limb: *F* (1, 11) = 160.3, *p* < 0.0001; limb-by-direction interaction: *F* (1.1, 11.9) = 117.8, *p* < 0.0001; SABD: *t* (11) = 3.9, *p* = 0.002; SADD: *t* (11) = 5.6, *p* = 0.0002; EF: *t* (11) = 6.7, *p* < 0.0001; EE: *t* (11) = 3.9, *p* = 0.003; WF: *t* (11) = 7.9, *p* < 0.0001; FF: *t* (11) = 6.6, *p* < 0.0001; Grip: *t* (11) = 11.7, *p* < 0.0001), and none of the paretic limbs could generate appreciable wrist or finger extension torque. There were no overall differences between the non-paretic and control limbs (main effect of limb: *F* (1, 16) = 1.8, *p* = 0.20; limb-by-direction interaction: *F* (8, 128) = 0.49, *p* = 0.86).

### Voluntary vs. synergy-driven maximal torque and muscle activation at proximal vs. distal joints

We predicted that the voluntary activation deficit and the voluntary strength deficit would differ among muscles and torque directions, with low values for proximal muscles and torque directions and higher values for distal muscles and torque directions.

**Figure 2:**
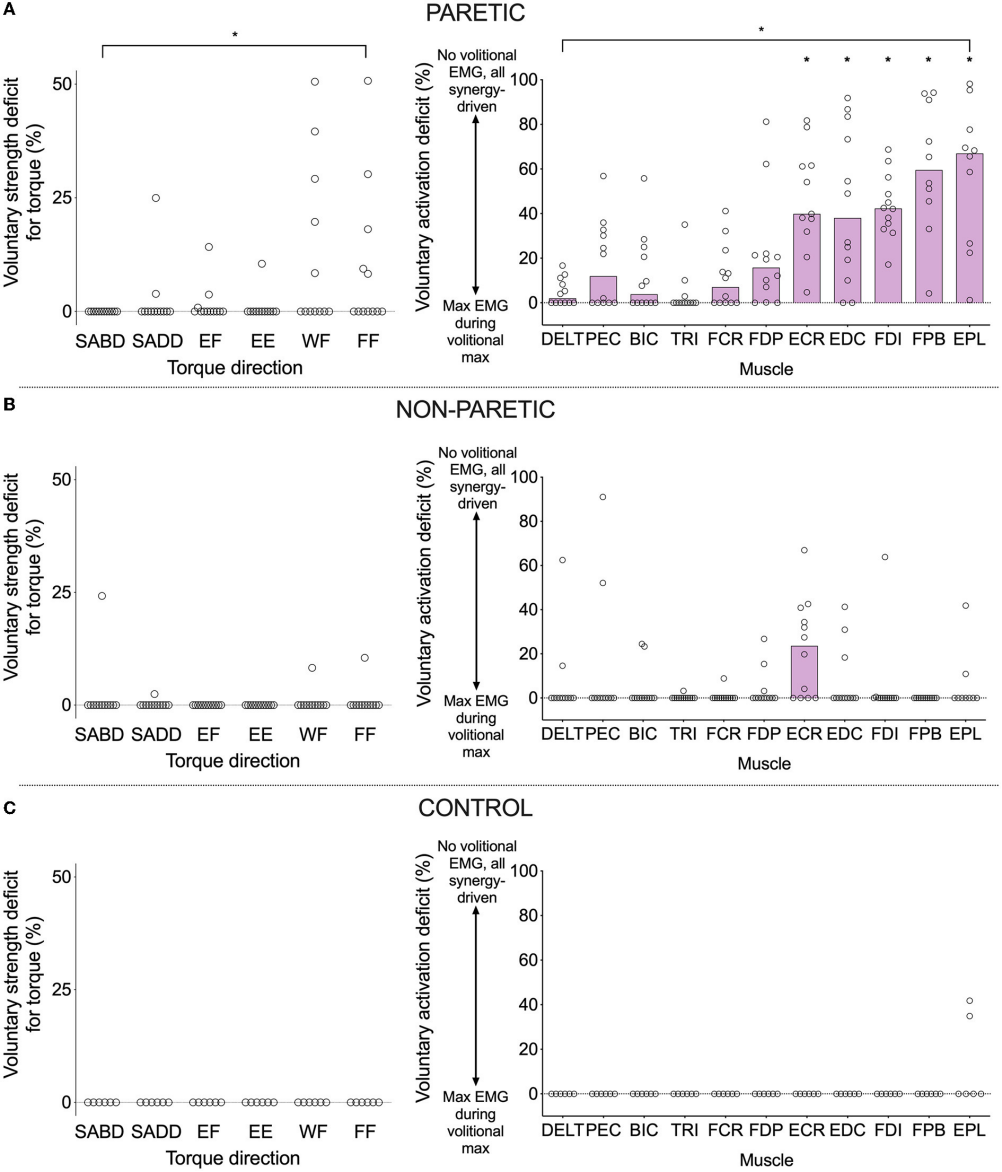
Voluntary strength deficit in torque (left) and voluntary activation deficit in EMG (right) for the paretic **(A)**, non-paretic **(B)**, and control **(C)** limbs. A value of zero indicates that maximal torque/EMG was obtained during a contraction in the intended/agonist direction, and non-zero values indicate that maximal torque/EMG was obtained during a contraction in a different direction (i.e., synergy-driven). The higher the value, the larger the discrepancy between the voluntary and synergy-driven values. Circles indicate values for individual participants, and purple bars indicate the group median (lack of a purple bar indicates a group median of zero). Asterisks indicate significant differences between values for a particular muscle and those of the deltoid at *p* ≤ 0.05 (*). Exact *p*-values are presented in the text.

For the voluntary activation deficit in EMG, our results were in harmony with this prediction. The values differed among the 13 muscles (Figure 2B) (significant effect of muscle (*F* (10, 105) = 11.6, *p* < 0.0001). Group means were higher for wrist/finger extensors (*ECR*: 46.1%, *t* (105) = 4.6, *p* < 0.0001; *EDC*: 46.1%, *t* (105) = 4.7, *p* < 0.0001) and intrinsic hand muscles (*FDI*: 46.3%, *t* (105) = 4.8, *p* < 0.0001; *FPB*: 60.4%, *t* (105) = 6.1, *p* < 0.0001; *EPL*: 59.2%, *t* (105) = 6.0, *p* < 0.0001) compared with the deltoid (4.8%). Values for the remaining muscles, including the wrist/finger flexors, were not significantly different from those of the deltoid [*PEC*: 18.3%, *t* (105) = 1.6, *p* = 0.12; *BIC*: 12.3%, *t* (105) = 0.9, *p* = 0.40; *TRI*: 4.0%, *t* (105) = 0.1, *p* = 0.92; *FCR*: 11.5%, *t* (105) = 0.8, *p* = 0.44; *FDP*: 21.4%, *t* (105) = 1.9, *p* = 0.06]. Similarly, the voluntary strength deficit for torque differed among primary torque directions (Figure 2A) [significant effect of primary torque direction (Friedman statistic = 12.5, *p* = 0.029)].

However, when comparing the voluntary strength deficit for SABD (0%) with that of the other primary torque directions in a pairwise fashion, there were no significant differences (*SADD*: rank sum difference = −5.0, *z* = 0.55, *p* = 0.59; *EF*: rank sum difference = −8.0, *z* = 0.87, *p* = 0.38; *EE*: rank sum difference = −2.5, *z* = 0.27, *p* = 0.79; *WF*: rank sum difference = −16.0, *z* = 1.74, *p* = 0.08; *FF*: rank sum difference = −16.5, *z* = 1.8, *p* = 0.07). Nonetheless, there are notable findings worthy of discussion that are in line with our prediction. Eight of the 12 participants generated more paretic torque during a synergy-driven contraction than during a voluntary contraction (i.e., a non-zero voluntary strength deficit) for at least one primary torque direction (Table 2 and Figure 2A). SABD was the only direction in which all participants generated maximal torque during voluntary contraction. For the remaining contraction directions, the number of participants with maximal torque during the voluntary contraction was fewer at the wrist and finger joints than shoulder and elbow joints (SADD: 10, EF: 9, EE: 11, WF: 7, FF: 7). For the participants whose maximum torque was achieved via synergy-driven rather than voluntary efforts, the discrepancy between the voluntary and synergy-driven activation was substantially greater for the wrist and finger joints, with the voluntary strength deficit values averaging 14.4, 6.2, 10.5, 29.5, and 23.3% for the SADD, EF, EE, WF, and FF directions, respectively. The specific synergy-driven direction that produced a higher torque value for a given primary torque direction was within the flexion or extension synergy pattern (e.g., maximal EF torque was produced during SABD and maximal EE torque was produced during SADD; Table 2).

For the non-paretic and control limbs (Figures 2B,C), the voluntary strength deficit and voluntary activation deficits were zero in the vast majority of cases. However, there was more variability in the non-paretic data compared with the control data. Most notably, eight of the 12 participants had a non-zero voluntary activation deficit for the ECR muscle. For most of these participants, the maximum EMG value for ECR occurred during the generation of maximal EF torque, which is not surprising given that ECR has a small flexion moment about the elbow.

### Proximal vs. distal elicitation of the flexion and extension synergies

**Figure 3:**
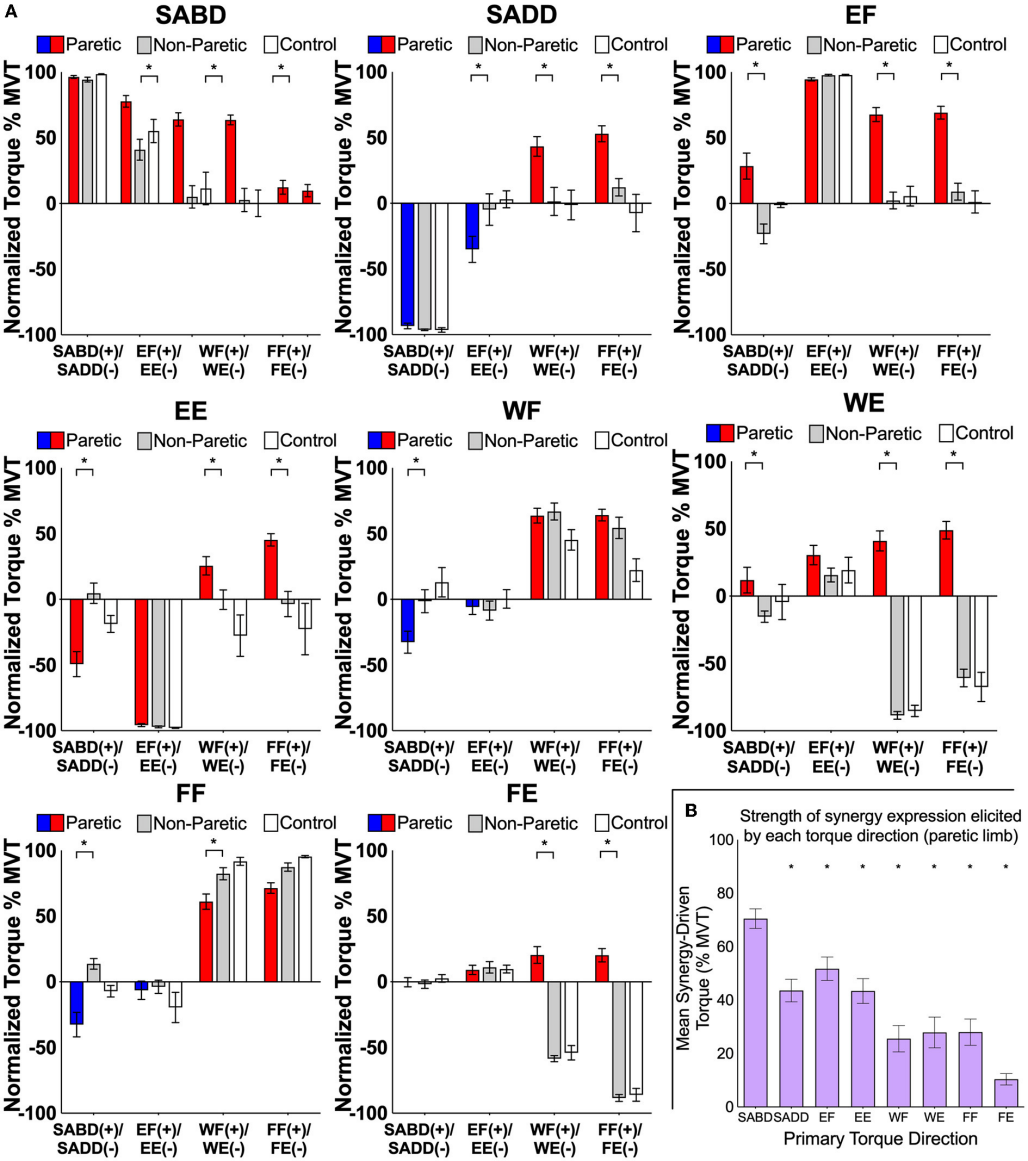
**(A)** Group mean ± SEM shoulder, elbow, wrist, and finger joint torques produced by the paretic (red/blue), non-paretic (gray), and control (white) limbs during the generation of SABD, SADD, EF, EE, WF, WE, FF, and FE MVT. For the paretic limb, red bars highlight SABD and flexion directions, and blue bars highlight SADD and extension directions for the paretic limb. Asterisks indicate significant differences between paretic and non-paretic limbs (*p-*values range from < 0.0001 to 0.01) from our planned comparisons on the limb-by-secondary torque interactions. Non-paretic and control data were similar with no meaningful variations. Normalized torques in wrist flexion and extension for the non-paretic and control data that are not near maximum resulted because maximal torque values often occurred during combined rather than the wrist and finger flexion or extension. These findings are related to the WFTS device and positioning and are not believed to have scientific relevance. **(B)** The mean synergy-driven torque generated by each primary torque direction for the paretic limb. This was calculated by averaging the magnitude of all secondary torques generated during the performance of a given primary torque direction. Asterisks indicate that synergy-driven torque for a given primary torque direction was significantly lower value than that for SABD (*p-*values range from < 0.0001 to 0.02; exact values are reported in the text).

First, we show that expression of the flexion and extension synergies by the paretic limb is different from the biomechanical coupling among joints of the non-paretic and control limbs during maximal contractions [as has been shown previously for SABD, SADD, EF, and EE torque directions (1, 3)] (Figure 3A). Secondary torques of the paretic limb differed from those of the non-paretic limb for all eight primary torque directions (significant limb-by-secondary torque direction interactions: SABD: *F* (3, 33) = 15.4, *p* < 0.0001; SADD: *F* (3, 33) = 17.5, *p* < 0.0001; EF: *F* (3, 33) = 17.7, *p* < 0.0001; EE: *F* (3, 33) = 31.0, *p* < 0.0001; WF: *F* (3, 33) = 5.3, *p* = 0.004; WE: *F* (3, 33) = 47.1, *p* < 0.0001; FF: *F* (3, 33) = 5.3, *p* = 0.004; FE: *F* (3, 33) = 94.8, *p* < 0.0001). Non-paretic and control data were similar with no meaningful variations.

Second, in terms of our prediction that the generation of torque in proximal primary torque directions would result in stronger synergy expression than the generation of torque in distal primary torque directions, we found that the magnitude of synergy expression differed among primary torque directions (Figure 3B, significant effect of primary torque direction, *F* (7.77) = 24.7, *p* < 0.0001). It was stronger for SABD (68.1 ± 3.7% MVT) than for each of the other primary torque directions (*SADD*: 44.9 ± 4.1% MVT, *t* (77) = 4.3, *p* < 0.0001; *EF*: 57.0 ± 4.0% MVT, *t* (77) = 2.1, *p* = 0.04; *EE*: 42.7 ± 3.8% MVT, *t* (77) = 4.7, *p* < 0.0001; *WF*: 25.6 ± 4.9% MVT, *t* (77) = 7.9, *p* < 0.0001; *WE*: 27.9 ± 5.8% MVT, *t* (77) = 7.5, *p* < 0.0001; *FF*: 28.0 ± 4.9% MVT, *t* (77) = 7.5, *p* < 0.0001; *FE*: 10.4 ± 2.1% MVT, *t* (77) = 10.8, *p* < 0.0001).

**Figure 4:**
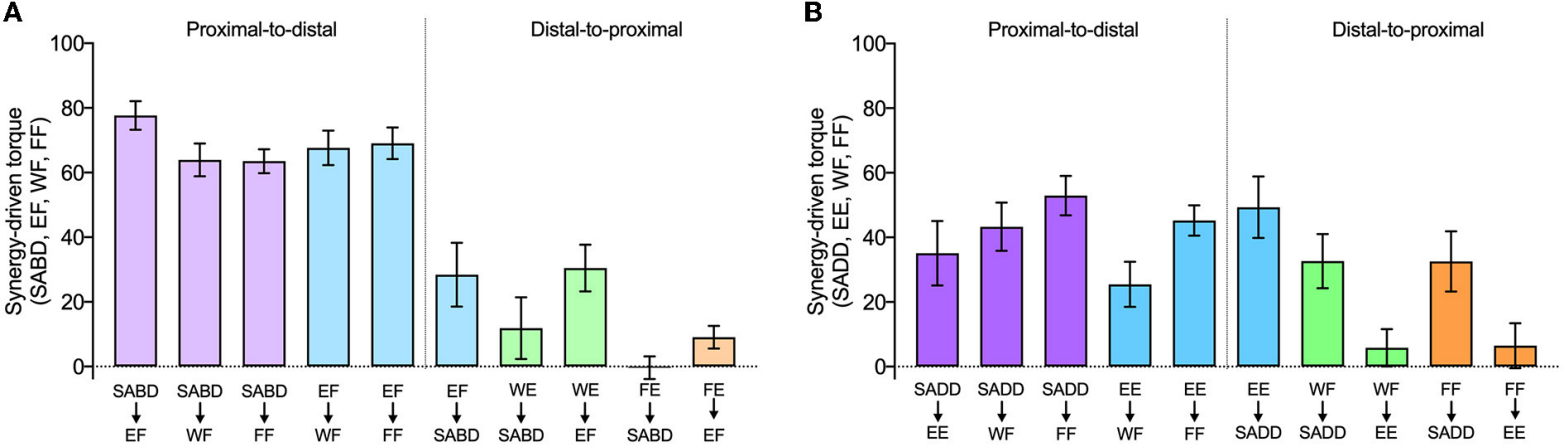
The strength of proximal-to-distal vs. distal-to-proximal elicitation of the flexion and extension synergies. Group mean ± SEM synergy-driven torques (the same as in Figure 3) are shown for each joint combination and direction, as indicated on the x-axis. The top row of labels is the primary torque direction, and the bottom row of labels is the secondary torque direction. Data from primary torque directions that elicited the flexion synergy are shown in **(A)**, and data from primary torque directions that elicited the extension synergy are shown in **(B)**. Note that attempts to generate WE and FE (i.e., when they were the primary torque directions) instead resulted in the production of WF and FF, respectively.

In the following paragraphs, we present details of synergy expression for each primary torque direction and joint combination. Generation of maximal SABD and EF by the paretic limb both resulted in secondary torques that were consistent with expression of the flexion synergy (EF, WF, FF during SABD; SABD, WF, FF during EF), and there was a difference among the joint combinations (significant effect of joint combination, *F* (9, 110) = 22.8; *p* < 0.0001) (Figure 4, left panel). The shoulder-to-elbow effect of the flexion synergy was stronger than the elbow-to-shoulder effect. Generation of maximal SABD induced more secondary EF torque (77.7 ± 4.4% EF MVT) than the generation of maximal EF induced secondary SABD torque (28.4 ± 9.9% SABD MVT) (*t* (110) = 5.7, *p* < 0.0001). Generation of SABD and EF produced similar amounts of secondary WF torque (64.0 ± 5.1% and 67.7 ± 5.3% MVT, respectively, *t* (110) = 0.43, *p* = 0.67) and secondary FF torque (63.5 ± 3.7% and 69.1 ± 4.9% MVT, respectively, *t* (110) = 0.64, *p* = 0.52).

During the intended generation of maximal WE and FE torques, the paretic limb group instead produced torque in the WF and FF directions, as we anticipated based on previous literature. Efforts to produce WE torque elicited the flexion synergy in the paretic limb, as evidenced by SABD and EF secondary torques that were small to moderate in magnitude (11.9 ± 9.5% and 30.5% ± 7.2% MVT, respectively). When FE was the primary torque direction, however, virtually no torque was produced at the shoulder for either limb group, and the amount of EF torque was similar between groups. Similar to the shoulder and elbow comparisons, proximal-to-distal elicitation of the flexion synergy led to stronger secondary torques than distal-to-proximal elicitation (SABD led to stronger WF (64.0%) than WE led to SABD (11.9%), (*t* (110) = 6.0; *p* < 0.0001). The same pattern was seen between the elbow and wrist and elbow and fingers. Maximal EF torque led to greater secondary WF torque (67.7 ± 5.3% MVT) than maximal WE led to secondary EF torque (30.5 ± 7.2% MVT (*t* (110) = 4.3, *p* < 0.0001), and it led to greater secondary FF torque (69.1 ± 4.9% MVT) than maximal FE led to secondary EF torque [9.1 ± 3.5% MVT, (*t* (110) = 6.9, *p* < 0.0001)].

Generation of SADD and EE demonstrated secondary torques that were consistent with expression of the extension synergy (EE, WF, FF during SADD; SADD, WF, FF during EE), and there was a significant difference among the joint combinations (significant effect of joint combination, *F* (9, 110) = 4.6, *p* < 0.0001) (Figure 4, right panel). Unlike the flexion synergy, however, the magnitude of extension synergy expression elicited via the shoulder was not stronger than that elicited via the elbow. Secondary EE torque produced during maximal SADD (49.3 ± 9.5% EE MVT) was not different than secondary SADD torque produced during maximal EE (35.1 ± 9.9% SADD MVT) (*t* (110) = 1.3, *p* = 0.19). Secondary WF torque produced during maximal SADD (42.2 ± 7.1% WF MVT) was not different than that produced during maximal EE (25.5 ± 7.0% WF MVT) (*t* (110) = 1.6, *p* = 0.10), and secondary FF torque produced during maximal SADD (52.7 ± 6.2% FF MVT) was not different than that produced during maximal EE (46.7 ± 4.4% FF MVT (*t* (110) = 0.7, *p* = 0.48). During the generation of WF and the generation of FF, the paretic limb produced 32.6 ± 8.4% and 32.6 ± 9.3% MVT of secondary SADD torque, respectively. There was not a difference in extension synergy expression when examining proximal-to-distal vs. distal-to-proximal elicitation between SADD and WF (*t* (110) = 0.98, *p* = 0.33) or between SADD and FF (*t* (110) = 1.87, *p* = 0.06).

There was no appreciable secondary elbow torque produced during WF or FF, although the generation of torque in these directions elicited the extension synergy pattern in other degrees of freedom at the shoulder and the forearm, evidenced by shoulder flexion, shoulder internal rotation, and forearm pronation torques that were measured but are not presented in this study. The difference in extension synergy expression when examining proximal-to-distal vs. distal-to-proximal elicitation between EE and WF was not significant (*t* (110) = 1.81, *p* = 0.07). However, for this comparison between EE and FF, proximal-to-distal elicitation was greater than distal-to-proximal elicitation (*t* (110) = 3.57, *p* = 0.0005).

### Proximal vs. distal elicitation of contralateral associated reactions

**Figure 5:**
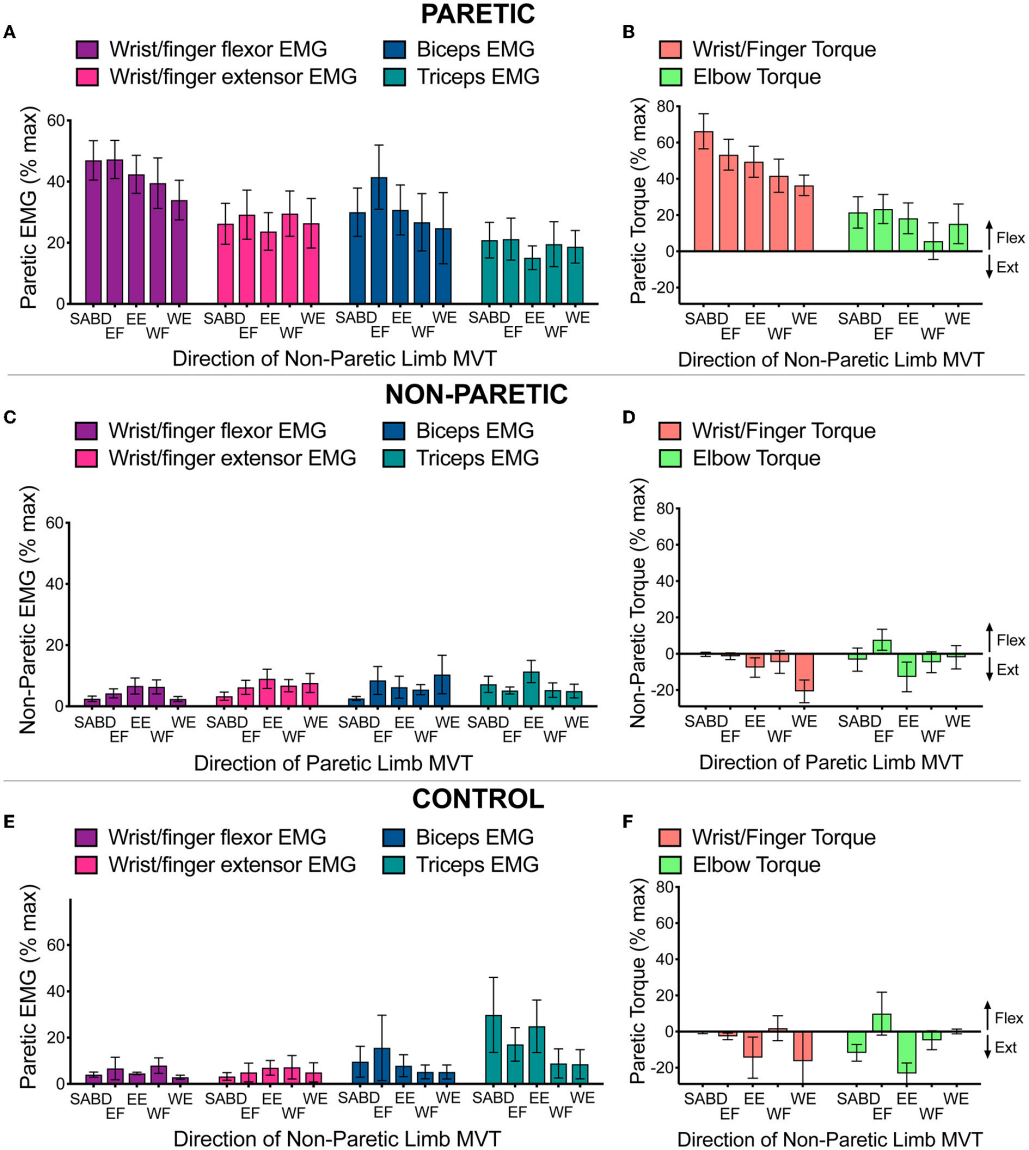
Voluntary strength deficit in torque **(A,C,E)** and voluntary activation deficit in EMG **(B,D,F)** for the paretic **(A,B)**, non-paretic **(C,D)**, and control **(E,F)** limbs during maximal voluntary efforts by the contralateral limb in SABD, EF, EE, WF, and WE directions. Values shown are based on the actual data, not the linear mixed effect model estimated values that are presented in the text. Outlier elbow torque data from the paretic limb of one participant who demonstrated near maximal levels of elbow extension torque is not shown (see text for details).

**Figure 6:**
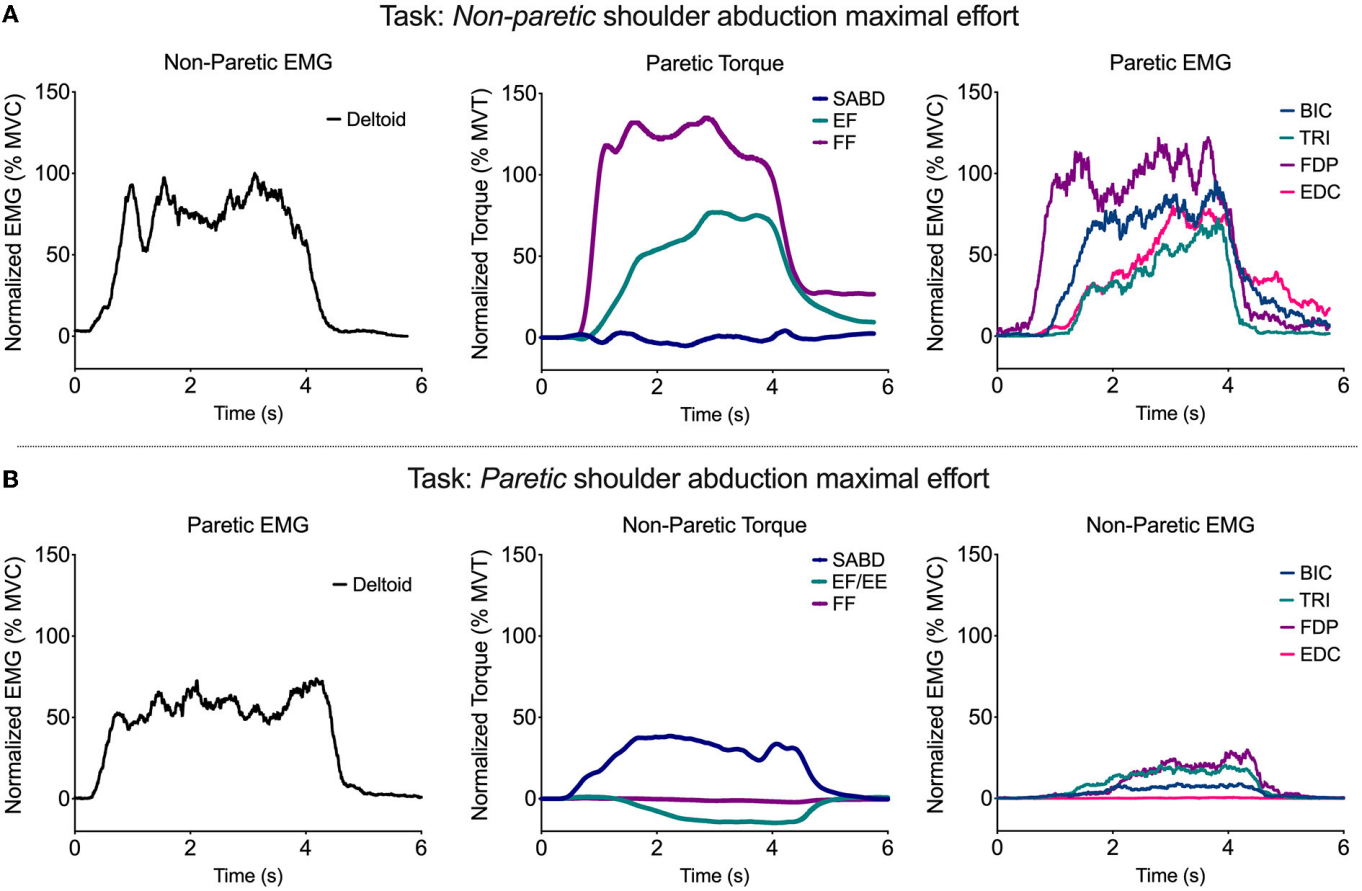
Representative single-trial data from one participant during a shoulder abduction maximal effort performed by the non-paretic **(A)** and paretic **(B)** limbs. EMG from the deltoid of the limb performing the task is shown in the left plot, SABD, EF/E, and FF torque from the contralateral limb is shown in the middle plot, and BIC, TRI, FDP, and EDC EMG from the contralateral limb are shown in the right plot.

First, we show that maximal activation of contralateral muscles produced stronger contractions in the paretic limb than in the non-paretic or control limbs (Figures 5, 6). This was the case for all muscle groups and was most notable in the wrist/finger flexors (averages across contraction directions: *wrist/finger flexors*: 41.6 ± 3.1% MVC vs. 3.8 ± 3.1% MVC; significant main effect of limb: *F* (1, 85.0) = 177.0, *p* < 0.0001; *wrist/finger extensors*: 26.3 ± 3.1%, MVC vs. 6.6 ± 3.1% MVC; significant main effect of limb: *F* (1, 86.8) = 38.3, *p* < 0.0001; *BIC*: 29.1 ± 4.0% MVC vs. 5.2 ± 4.0% MVC; significant main effect of limb: *F* (1, 85.9) = 39.5, *p* < 0.0001; *TRI*: 20.8 ± 2.5 vs. 6.4 ± 2.5% MVC; significant main effect of limb: *F* (1, 87.0) = 28.4, *p* < 0.0001). For the non-paretic and control limbs, maximal activation of contralateral muscles produced low levels of co-contraction of flexors and extensors at both the wrist/fingers and elbow that were similar between groups.

For 10 out of 12 participants, there was at least one paretic wrist/finger EMG and/or torque value produced during contralateral torque generation that was higher than the maximal value produced in the paretic limb during ipsilateral torque generation at any of the joints. This occurred most frequently in the wrist and finger torque directions and muscles [elbow extension torque (one participant), wrist flexion torque (two participants), finger flexion torque (three participants), PEC EMG (two participants), BIC EMG (one participant), FCR EMG (one participant), FDP EMG (two participants), ECRb EMG (two participants), FDI EMG (five participants), FPB EMG (1 participant), and EPL EMG (1 participant)]. On average across these occurrences, the maximal torque or EMG value produced during contralateral contractions was 145% of the maximal value produced during ipsilateral contractions (range: 103–485%).

Second, we address our prediction that activation of proximal muscles in the non-paretic arm would result in stronger associated reactions in the paretic arm compared with activation of distal muscles in the non-paretic arm. Our findings were in support of this prediction for the wrist/finger flexors but not for the biceps, triceps, or wrist/finger extensors. Further, we predicted that associated reactions in the paretic arm would be stronger in flexor muscles compared with extensor muscles. Our findings are in support of this prediction for the wrist/finger muscles but not the elbow muscles.

Specifically, the contralateral contractions activated wrist/finger flexor muscles more strongly on average than wrist/finger extensor muscles (41.4 ± 5.4% vs. 25.5 ± 6.2% MVC, significant main effect of the muscle group, *F* (1, 10.0) = 9.3, *p* = 0.01; note that these means differ very slightly from those presented in the preceding paragraph because they are marginal means estimated from each statistical model, not the means of the underlying data). In addition, contralateral contractions in the various directions activated the muscle groups differently (significant muscle group-by-contraction direction interaction: *F* (4, 48.3) = 2.9, *p* = 0.03). The wrist/finger flexors demonstrated a decreasing pattern of activation when comparing directions from proximal to distal and the wrist/finger extensors demonstrated an overall consistent pattern of activation among contralateral contraction directions. In accordance with the EMG data, contralateral contractions resulted in a substantial amount of paretic wrist and finger flexion torque that differed among contraction directions in a decreasing manner from proximal to distal (significant effect on contraction direction (*F* (4, 31.1) = 3.4, *p* = 0.02); group mean values ranging from 66.2 ± 9.7% MVT for the shoulder abduction direction to 36.4 ± 5.7% MVT for the wrist extension direction).

For the paretic biceps and triceps, the paretic elbow flexor and extensor muscles were activated at similar levels to each other [28.7 ± 7.2% vs. 20.4 ± 4.8% MVC for BIC and TRI, respectively; no significant main effect of muscle (*F* (1, 10.2) = 1.75, *p* = 0.22); no significant muscle-by-contraction direction interaction (*F* (4, 59.1) = 1.59, *p* = 0.19)], which is in contrast to the pattern seen in wrist/finger muscles. For paretic elbow torque, contralateral contractions resulted largely in flexion torque (i.e., eliciting the flexion synergy) for all but one participant, who produced maximal levels of elbow extension torque as part of the extension synergy. There was no effect on contraction direction (*F* (4, 32.4) = 0.21, *p* = 0.93). For all participants, paretic elbow flexion torque averaged 8.8 ± 9.8% MVT over contraction directions, with no significant effect on contraction direction. When excluding the participant who exhibited the strong extension synergy, paretic elbow flexion torque averaged 16.7 ± 6.6% MVT.

## Discussion

Our primary findings are that (1) wrist and finger muscles are often activated more strongly during maximal synergy-driven contractions than during maximal voluntary contractions, (2) expression of the flexion and extension synergies is strongest when elicited via proximal rather than distal muscle contractions, and (3) associated reactions in the paretic wrist/finger flexors were stronger than those of other paretic muscles and were the only ones whose response had a proximal to distal decreasing pattern. We interpret our findings as being consistent with an increased influence of brainstem motor pathways, based on the similarities between the effects we saw and the neuroanatomy and activation patterns of this system.

### Maximal synergy-driven contractions can be higher than maximal voluntary contractions, particularly for extrinsic wrist/finger extensors and intrinsic hand muscles

We predicted that maximal activation of proximal paretic muscles (i.e., those of the shoulder and elbow) would be achieved through voluntary contractions but that maximal activation for the most distal paretic muscles (i.e., those of the wrist and fingers) would occur during synergy-driven contractions. We reasoned that this finding would be consistent with the ways in which the muscles are impacted by stroke-induced corticospinal and corticobulbar tract damage and the increased reliance on brainstem motor pathways that follows. For example, with corticospinal damage, distal paretic muscles lose more of the neural substrate typically used for voluntary activation compared with proximal muscles, but they can still be activated by brainstem pathways via synergy-driven activation.

Using the voluntary activation deficit to quantify how maximal synergy-driven contractions compare to maximal voluntary contractions, we found that extrinsic wrist/finger extensors (ECR, EDC) and intrinsic hand muscles (FDI, FPB, EPL) had the largest and most frequently occurring increase in synergy-driven activation compared with voluntary activation, which is in line with our prediction. Voluntary activation was only 48% of the synergy-driven activation for those muscles on average, with virtually all participants demonstrating a non-zero voluntary activation deficit. In contrast to our prediction, voluntary activation deficit values for the extrinsic wrist/finger flexors were not statistically different than that for the DELT. The difference in the findings between the wrist/finger flexors and the wrist/finger extensors likely reflects the fact that in the intact nervous system, brainstem motor pathways facilitate distal flexors to a greater degree than extensors (16, 21), and the strength of this facilitation becomes greater following stroke (37). Further, while corticospinal projections are strong to all distal muscles, they are stronger in intrinsic hand muscles and distal extensors compared with distal flexors (19, 38). Thus, following corticospinal damage, it appears that shoulder and elbow muscles and wrist/finger flexors can still be activated voluntarily using brainstem pathways as well as remaining corticospinal resources, whereas wrist/finger extensors and intrinsic hand muscles rely primarily on remaining corticospinal resources.

While the persistence of brainstem pathways following stroke afford the shoulder, the elbow, and the wrist/finger flexors the ability to have some remaining voluntary activation, we must point out that the notable consequence of utilizing predominantly brainstem pathways is the loss of independent joint control that occurs when the flexion and extension synergies are expressed (6, 8–10).

### Flexion and extension synergy expression is strongest when the synergies are elicited via proximal rather than distal muscle contractions

Because of the aforementioned bias of innervation by brainstem pathways toward proximal muscles, we predicted that activation of these muscles would result in stronger synergy expression when compared with activation of distal muscles. Indeed, our findings support this prediction. When comparing synergy elicitation from one joint to another and vice versa, the proximal-to-distal elicitation was larger than the distal-to-proximal elicitation for every comparison (except for SADD and EE, for which the elicitation was not different between the directions).

When brainstem pathways are activated with the intent to drive shoulder muscles, the elbow and hand are activated as well-due to the system's diffuse multi-joint projections. In the intact nervous system, this multi-joint activation may be utilized for postural adjustments and/or to provide multi-joint stability, but the corticospinal tract and its cortico-reticular projections can selectively “gate” or inhibit reticulospinal effects at other joints when they are unwanted (23, 39). Following a stroke, however, unwanted effects of brainstem pathways at muscles of other joints are not suppressed, and the flexion and extension synergy patterns emerge. Our findings suggest that the strength of the brainstem pathway influence on muscles in one joint determines how strongly the synergy is elicited in muscles of other joints.

### Associated reactions are strongest when elicited via proximal rather than distal muscle contractions, but only in the wrist and finger flexors

As expected, we observed strong associated reactions in the paretic limb (unintended activation of paretic muscles that occurred during maximal contractions of the non-paretic limb). We predicted that the associated reactions would be stronger with proximal rather than distal contractions. We found this to be the case for the paretic wrist and finger flexors. Strong wrist/finger flexion torque was produced for all contraction directions, but it was lower when the contralateral joint was more distal, decreasing by nearly 50% when comparing torque resulting from contralateral shoulder abduction to that of contralateral wrist extension. The proximal-distal decreasing pattern in flexion torque across contraction directions was driven by selective decreases in wrist/finger flexor EMG rather than overall decreases in EMG for both flexor and extensor groups. Although not dependent on contraction direction, there was still an appreciable amount generated during the wrist extension contraction direction. Interestingly, however, paretic elbow torque did not depend on non-paretic contraction direction, evidenced by levels of elbow flexion torque that were consistent across contraction directions and were milder in comparison to that of the wrist/fingers (aside from the one outlier who generated maximal levels of elbow *extension* torque).

The presence of substantial bilateral muscle activity during non-paretic contractions is consistent with the bilateral upper limb projections of the cortico-reticulospinal pathway. The finding that the wrist/finger flexor muscles (FCR, FDP, FDI) demonstrated the most pronounced activation was of interest, as was the dependence on whether the contraction direction was proximal or distal. These results are consistent with the work of Zaaimi et al. (37) who found that, following unilateral corticospinal lesions in non-human primates, reticulospinal connections strengthen selectively to wrist/finger flexors but not extensors. The bilateral organization of the reticulospinal tract has been shown to activate muscles as far distal as the wrist (16, 21, 22), but whether this bilateral organization extends to muscles acting on digits of the hand has not yet been investigated (40).

While it could be argued that increased activity of the bilaterally projecting reticulospinal tract would also cause associated reactions in the non-paretic limb during paretic limb activation, it is likely that the intact crossed corticospinal tract that projects to the non-paretic limb helps suppress such associated reactions.

### Implications for clinical research

Results of the study underscore the need to acknowledge whole-limb behavior when examining motor control of the post-stroke upper limb. Studies examining a joint in isolation from the rest of the limb should consider whether results will generalize to functional scenarios when proximal or distal muscles are concurrently activated. Although the current study examines paretic limb behavior during maximal rather than functional efforts, the involuntary coupling between joints via the flexion and extension synergy patterns also occurs at submaximal efforts (1), including those commensurate with lifting the limb against gravity (5).

Insight derived from previous studies quantifying flexion and extension synergy expression provided the foundation for a novel physical therapy intervention for reaching based on progressive shoulder abduction loading (41–43) and helped to improve the control of assistive technologies (44, 45) for the post-stroke upper limb. Results of the current study add to this body of empirical evidence. For example, knowledge of how movement of any of the four paretic upper limb joints (shoulder, elbow, wrist, and finger) elicits the multi-joint synergy patterns could inform the control of a technology that assists the limb differently based on the intended task.

Results suggest that physical therapy interventions using bilateral movements (46) or assistive technologies controlled with the non-paretic limb (47, 48) may elicit associated reactions in the paretic limb when the amount of effort to the non-paretic limb is high. This may be particularly evident with activation of proximal non-paretic muscles. Additionally, while bilateral training may have important benefits including alterations in intra-cortical inhibition (49), it may also upregulate ipsilateral cortico-reticulospinal connections. This would further compound the elicitation of associated reactions during non-paretic limb movement and elicitation of the flexion and extension synergies during paretic limb activation, leading to increased difficulty in controlling joints independently during functional tasks.

### Limitations

Several limitations to the study should be considered. First, the sample size was small; however, consistent results were seen in the majority of participants. Second, because measurements were taken during isometric contractions, the exact ways that they translate to movement and functional mobility of the arm are not clear. However, because previous flexion synergy quantification studies that utilized dynamic contractions have corroborated findings obtained from isometric contractions (4, 15, 41), it is likely that this would also be the case for our findings.

It would also be useful to investigate relationships between our findings and measurements of arm impairment, function, and/or motor performance. Because the majority of participants in our study had severe hemiparesis of the paretic arm and hand, we do not have a large enough range of impairment levels to appropriately investigate relationships between our torque/EMG measurements and FMA or CMSAh score. Fortunately, several previous studies have investigated associations between lab-based synergy measures and arm function/impairment (5, 41).

In our previous studies quantifying synergy expression, we have established that secondary torques in the paretic arm generated during voluntary shoulder and elbow muscle activation are not compensatory behaviors or biomechanical couplings that occur during maximal torque generation (i.e., they are demonstrably different than findings in the non-paretic and control limbs) (1, 3, 5, 11). The present study is the first to examine secondary torques generated by the voluntary wrist and finger muscle activation. Thus, we should consider whether these secondary torques were compensatory, particularly given the torque directions that were paired. For example, perhaps shoulder adduction occurred during wrist/finger flexion in a compensatory manner if the participant pushed the whole arm down attempting to assist maximal wrist/finger flexor torque. Similarly, perhaps shoulder abduction occurred in the same manner if the participant lifted the whole arm attempting to assist maximal wrist/extension. However, if either was the case, the same compensatory behaviors would be expected in non-paretic and control limbs, given that all contractions were maximal efforts and the difficulty between groups would be similar. Because they were found only in the paretic limb, it is likely that these proximal secondary torques that occur during maximal wrist and finger torque generation are related to obligatory synergy expression.

Additionally, it is possible that the effects of contralateral contractions on the tested limb might have been different if the contralateral arm were in a different position. The effects of paretic upper limb position on ipsilateral reflex behavior (50), strength generation (51), and extension synergy expression (13) have been previously demonstrated. In particular, the shoulder adduction/elbow extension coupling of the extension synergy was shown to switch to shoulder adduction/elbow flexion when the arm was placed closer to the body than in the current study (13). However, the influence of the non-paretic upper limb position on the paretic upper limb is unknown.

Finally, the neuroscientific implications drawn from the results are speculative given the behavioral nature of our measurements. However, they are consistent with recent work that has been able to probe the involvement of various neural circuits more directly (6, 8–10). They also rely on the premise that the motor deficits in our post-stroke participants result from damage to the lateral corticospinal tract. Indeed, the hemiparesis and loss of fractionated movements seen in our participants have long been attributed to the disruption of pre-decussation corticospinal fibers (52–54). Further, two recent studies that examined white matter integrity of descending motor pathways in the sub-cortical region (10) and the brainstem (6, 10) in participants with the same inclusion criteria as the present study demonstrated decreased white matter integrity in the ipsilesional corticospinal tract and increased white matter integrity in contralesional reticulospinal tracts. These changes were associated with synergy expression (10) and overall upper limb impairment (6).

## Data availability statement

The raw data supporting the conclusions of this article will be made available by the authors, without undue reservation.

## Ethics statement

All participants gave written informed consent to participate in the study, which was approved by the Institutional Review Board of Northwestern University.

## Author contributions

LM and JD conceived and designed the research, interpreted the data, edited and finalized the submitted manuscript, and agreed to be accountable for all aspects of the work. LM acquired and analyzed the data and prepared the initial manuscript. Both authors contributed to the article and approved the submitted version.

## Funding

Research reported in this publication was supported by the National Institute on Disability, Independent Living, and Rehabilitation Research grant H133G070089, the National Institutes of Health grants R01 HD039343, R01 NS105759, and T32 HD057845, the National Center for Advancing Translational Sciences of the National Institutes of Health under Award Number KL2 TR002346, the Northwestern University Feinberg School of Medicine, and a Promotion of Doctoral Studies (PODS) II Scholarship from the Foundation for Physical Therapy Research, Inc.

## Conflict of interest

The authors declare that the research was conducted in the absence of any commercial or financial relationships that could be construed as a potential conflict of interest.

## Publisher's note

All claims expressed in this article are solely those of the authors and do not necessarily represent those of their affiliated organizations, or those of the publisher, the editors and the reviewers. Any product that may be evaluated in this article, or claim that may be made by its manufacturer, is not guaranteed or endorsed by the publisher.

## Author disclaimer

The content is solely the responsibility of the authors and does not necessarily represent the official views of the National Institutes of Health or other funding agencies.
